# Blood Levels of Free-Circulating Mitochondrial DNA in Septic Shock and Postsurgical Systemic Inflammation and Its Influence on Coagulation: A Secondary Analysis of a Prospective Observational Study

**DOI:** 10.3390/jcm9072056

**Published:** 2020-06-30

**Authors:** Emmanuel Schneck, Fabian Edinger, Matthias Hecker, Natascha Sommer, Oleg Pak, Norbert Weissmann, Andreas Hecker, Martin Reichert, Melanie Markmann, Michael Sander, Christian Koch

**Affiliations:** 1 Department of Anesthesiology, Operative Intensive Care Medicine and Pain Therapy, University Hospital of Giessen and Marburg, 35392 Giessen, Germany; emmanuel.schneck@chiru.med.uni-giessen.de (E.S.); Melanie.Markmann@chiru.med.uni-giessen.de (M.M.); michael.sander@chiru.med.uni-giessen.de (M.S.); christian.koch@chiru.med.uni-giessen.de (C.K.); 2 German Center of Infection Research, Partner Site Giessen/Marburg/Langen, 35392 Giessen, Germany; 3 Excellence Cluster Cardiopulmonary Institute, Member of the German Center for Lung Research, Universities of Giessen and Marburg Lung Center, 35392 Giessen, Germany; matthias.hecker@innere.med.uni-giessen.de (M.H.); Natascha.Sommer@innere.med.uni-giessen.de (N.S.); Oleg.Pak@innere.med.uni-giessen.de (O.P.); Norbert.Weissmann@innere.med.uni-giessen.de (N.W.); 4 Department of General and Thoracic Surgery, University Hospital of Giessen, 35392 Giessen, Germany; Andreas.Hecker@chiru.med.uni-giessen.de (A.H.); martin.reichert@chiru.med.uni-giessen.de (M.R.)

**Keywords:** sepsis, DAMPs, SIRS, inflammation, coagulation, coagulopathy

## Abstract

Major surgery is regularly associated with clinical signs of systemic inflammation, which potentially affects the rapid identification of sepsis. Therefore, this secondary analysis of an observational study aims to determine whether NADH dehydrogenase 1 (ND1) mitochondrial DNA (mtDNA) could be used as a potential biomarker for the discrimination between septic shock and postsurgical systemic inflammation. Overall, 80 patients were included (septic shock (*n* = 20), cardiac artery bypass grafting (CABG, *n* = 20), major abdominal surgery (MAS, *n* = 20), and matched controls (CTRL, *n* = 20)). Quantitative PCR was performed to measure ND1 mtDNA. Thromboelastography was used to analyze the coagulatory system. Free-circulating ND1 mtDNA levels were significantly higher in septic shock patients compared to patients suffering from post-surgical inflammation ({copies/µL}: CTRL: 1208 (668–2685); septic shock: 3823 (2170–7318); CABG: 1272 (417–2720); and MAS: 1356 (694–2845); CTRL vs. septic shock: *p* < 0.001; septic shock vs. CABG: *p* < 0.001; septic shock vs. MAS: *p* = 0.006; CABG vs. MAS: *p* = 0.01). ND1 mtDNA levels in CABG patients showed a strong positive correlation with fibrinogen (correlation coefficient [*r*]= 0.57, *p* < 0.001) and fibrinogen-dependent thromboelastographic assays (maximum clot firmness, EXTEM: *r* = 0.35, *p* = 0.01; INTEM: *r* = 0.31, *p* = 0.02; FIBTEM: *r* = 0.46, *p* < 0.001). In conclusion, plasma levels of free-circulating ND1 mtDNA were increased in septic shock patients and were discriminative between sepsis and surgery-induced inflammation. Furthermore, this study showed an association between ND1 mtDNA and a fibrinogen-dependent pro-coagulatory shift in cardiac surgical patients.

## 1. Introduction

Sepsis is a hazardous complication that can follow major abdominal and cardiovascular surgery, and is associated with increased morbidity and mortality [1]. Because major surgery is regularly associated with clinical signs of systemic inflammation (e.g., fever, tachycardia, and altered mental status), the rapid identification of sepsis can be difficult. Furthermore, established biomarkers of inflammation cannot sufficiently discriminate septic conditions from regular post-surgical inflammation, leaving the clinician in a challenging situation [2].

Over the last decade, neutrophil extracellular traps (NETs) have been identified as major players in the development of septic shock and postsurgical systemic inflammation [3,4]. Released by neutrophil granulocytes, NETs consist of histones, myeloperoxidase (MPO), neutrophil elastase, platelets, and nucleic acids, such as mitochondrial DNA (mtDNA) [3]. Once released into the vascular system, NETs form web-like structures to trap pathogens, but can also snare platelets, resulting in the activation of the coagulatory system [5,6]. Furthermore, the plasmatic coagulatory system may be stimulated by the NET’s polyanionic surfaces and direct tissue factor presentation [6,7].

The primary study on which this secondary analysis is based aimed to identify NETs, measured by flow cytometry, as a potential discriminative biomarker between sterile and septic inflammation [8]. However, although NETs significantly increased after major surgery and during sepsis, the blood levels of free-circulating NETs did not differ between these conditions. Additionally, an association between NETs and an anticoagulatory pattern was observed in patients suffering from postsurgical systemic inflammation, although the primary study demonstrated the well-known pro-coagulatory properties of NETs in septic shock patients.

Because NETs failed to discriminate between septic shock and postsurgical inflammatory responses, this secondary analysis focused on mitochondrial DNA (mtDNA) as a potential discriminatory biomarker. Compared with NETs and other nucleic acids, mtDNA might represent a reasonable target for differentiation between inflammatory conditions caused by varying origins (e.g., sterile vs. infectious causes). First, NETs are mainly released locally in capillaries, organs, and other tissues, whereas free-circulating mtDNA can be found systemically [5]. Second, mtDNA differs from nuclear DNA, due to an increased ability to induce inflammatory responses via Toll-like receptor 9 (TLR-9), which in turn is pivotal for neutrophil activation. Moreover, mtDNA release has also been associated with the TLR-9-dependent activation of platelets, suggesting the possibility that mtDNA may be able to activate the coagulatory system [9]. Lastly, elevated mtDNA levels have been associated with increased intensive care unit (ICU) mortality [10]. MtDNA may therefore be able to differentiate between varying inflammatory conditions, such as sepsis and postsurgical inflammatory responses. Although mtDNA is known to be released during trauma, major abdominal and cardiovascular surgery, and sepsis, direct comparisons among these patient cohorts remain lacking [3,11,12]. Due to the mitochondrial origins of NADH dehydrogenase 1 (ND1), the plasma levels of free-circulating ND1 mtDNA have been used as a surrogate marker for mitochondrial damage under inflammatory conditions, and were therefore chosen as the target for this secondary analysis [13,14,15].

The goal of this follow-up analysis was to quantify the plasma levels of free-circulating ND1 mtDNA associated with septic shock and surgery-induced systemic inflammation. Furthermore, we aimed to identify any potential in vivo interaction between plasma levels of ND1 mtDNA and the coagulatory system [8,16,17].

## 2. Experimental Section

### 2.1. Study Design

This study is a secondary analysis, based on a single-center, prospective, observational proof-of-concept study that included 80 patients at the University Hospital of Giessen [8]. The local ethics committee approved the primary study, as well as the secondary data analysis (Justus-Liebig-University of Giessen, trial code: 86/18), and both were registered in the German Clinical Trials Register (trial code: DRKS00013584). Both the original study and this secondary analysis were performed in accordance with the Helsinki Declaration, and all methods and results are presented in accordance with the Strengthening the Reporting of Observational Studies in Epidemiology (STROBE) guidelines.

Between October 2018 and March 2019, consecutive patients of legal age were enrolled in the study, after signing informed consent forms. If patients were not sui juris, consent was obtained from their legal representatives. Exclusion criteria included current pregnancy or nursing, age < 18 years, history of severe valvular heart and/or autoimmune disease, recent suffering of severe trauma, the need for immunomodulatory medication, hematological disease, any prior need for extracorporeal membrane oxygenation, or renal replacement therapy. Septic shock patients were enrolled according to the Sepsis-3 definition of septic shock [18]. Because no baseline parameters were available in septic patients, control patients (CTRL) were matched based on age, gender, and underlying health conditions (arteriosclerosis, cancerous diseases, renal insufficiency, and diabetes mellitus). These pre-existing diseases were chosen because they are associated with increased NET generation [19,20]. Since NETs represented the target parameter of the primary study, these conditions might have biased the study results, and were therefore matched in the control and septic shock group. Since these conditions are also connected to an impaired mitochondrial function, the matching strategy is also appropriate for this secondary analysis [21,22,23,24]. Visceral surgical patients underwent major abdominal surgery (MAS), such as Whipple’s procedure, oncological gastric or esophageal resection, or colectomy, whereas all cardiac surgical patients received coronary artery bypass graft (CABG) surgery. Surgical patients had to fulfill at least two criteria associated with the definition of systemic inflammatory response syndrome (SIRS), within 24 h after surgery [25].

### 2.2. Sample Processing

In surgical patients, blood was collected prior to and immediately after surgery, as well as 24 and 72 h post-operatively. In patients who suffered from septic shock, blood was first taken after admission to the ICU and then again after 24 and 72 h. Furthermore, blood was drawn only at a single time point in CRTL patients. Blood was collected in citrate tubes for thromboelastometry and hirundine tubes for whole-blood, ristocetin-induced, platelet impedance aggregometry, whereas ethylenediaminetetraacetic acid (EDTA) tubes were used for the flow cytometry, polymerase chain reaction (PCR), and enzyme-linked immunosorbent assay (ELISA). Plasma samples were stored at −80 °C and thawed only once for ELISA analyses. Clinical data were obtained from the patient data management system (IMESO GmbH, Giessen, Germany).

### 2.3. Quantitative Polymerase Chain Reaction

The quantification of ND1 mtDNA by quantitative PCR (qPCR) was performed as previously described [10,13]. First, blood was collected (in a 7.5 mL EDTA tube) and centrifuged to separate plasma (200 *g* for 10 min at room temperature). Afterwards, 100 µL plasma were diluted with 100 µL phosphate-buffered saline (PBS), and the mixture was centrifuged again at 5000 *g* (10 min at 4 °C). The supernatant was frozen at −20 °C. After thawing, the mtDNA was purified with a commercial purification kit, according to the manufacturer´s instructions (QIAquick PCR Purification Kit, Qiagen, Venlo, The Netherlands). Next, the samples were diluted 1:20 with nuclease-free, deionized–distilled H_2_O before qPCR analysis. A StepOnePlus cycler (Thermo Fisher, Waltham, MA, United States) was used to quantify ND1 mtDNA in all samples. The ND1 mtDNA primers used were as follows: “ND1 mtDNA FW: 5´-CCA CCT CTA GCC TAG CCG TTT A-3´” and “ND1 mtDNA RW: 5´-GGG TCA TGA TGG CAG GAG TAA T-3´” (synthesized by Eurofins, Luxembourg, Luxembourg).

Samples were quantified using the mean values of triplicate measurements. The results were converted to copies/µL, according to Chiu et al. [26], using a standard curve. A plasmid containing human ND1 mtDNA (OriGene Technologies, Rockville, MD, United States) was used to establish the standard curve. The number of plasmid copies was calculated by a NanoDrop 2000 spectrophotometer (Thermo Fisher Scientific). Serial dilutions of the corresponding copy number of plasmid (30–300,000 copies per PCR reaction) were used.

### 2.4. Flow Cytometry

The primary analysis successfully evaluated a novel flow cytometry-based method to quantify NETs, which has been described in detail within the publication of the primary analysis [8]. In brief, after the identification of CD15+ neutrophils (Pacific BlueTM anti-human CD15 antibody, BioLegend, San Diego, CA, United States), NETs were identified by the positive staining of myeloperoxidase (MPO, ab11729, Abcam, Cambridge, United Kingdom) and anti-H3-Histone antibody (Alexa Fluor 647 Anti-Histone, BioLegend, San Diego, CA, United States) within the CD15+ cell population, in red-cell lysis samples processed for flow cytometry (BD FACS Canto II with BD FACSDIVA software, version 6.1.3, Becton Dickinson, Franklin Lakes, USA). Details regarding the gating strategy are explained in the primary analysis. Data are presented as the percentage of NETs for all gated neutrophils.

### 2.5. ELISA

ELISA analyses were used to measure interleukin-8 (IL-8; Human IL-8/CXCL8 Quantikine HS ELISA, R&D Systems, Minneapolis, MN, United States), high mobility group protein B1 (HMGB1; Human HMGB1 ELISA Kit, Aviva Systems Biology, San Diego, CA, United States) and MPO (Human MPO Instant ELISA, eBioscience, Frankfurt, Germany). All analyses were performed according to the manufacturer’s instructions. An automated plate reader (Epoch, BioTek Instruments GmbH, Heilbronn, Germany) was used, and the probes were measured in accordance with their recommended absorbances (IL-8: 490 nm, HMGB1: 450 nm, MPO: 450 nm).

### 2.6. Inflammatory Parameters

Plasma levels of C-reactive protein (CRP) and procalcitonin (PCT), as well as the blood cell count were performed during clinical routines in the local laboratory of the university hospital of Giessen.

### 2.7. Coagulatory Analysis

For thromboelastography (ROTEM, Matel Medizintechnik, Hausmannstaetten, Austria) and whole-blood, ristocetin-induced platelet impedance aggregometry (Multiplate, Roche Diagnostics, Rotkreuz, Switzerland), point-of-care devices were used, and all other coagulatory tests were performed by the local clinical laboratory. Thrombelastographic assays included NATEM, INTEM, FIBTEM, and EXTEM. For each assay the clot formation time (CFT; seconds), clotting time (CT; seconds), mean clot firmness (MCF; mm), and lysis index after 60 min (LI60; %) was measured. For whole-blood, ristocetin-induced platelet impedance aggregometry, platelets were stimulated with ADP (ADPtests), thrombin-receptor activator protein 6 (TRAPtest), and arachidonic acid (ASPItest).

### 2.8. Statistical Analysis

Values were tested for normal distribution using the Shapiro–Wilk test. Parametric data were expressed as the mean and standard deviation, whereas the median and interquartile range (IQR) were used for non-parametric data. To identify a potential interaction between plasma levels of free-circulating NETs and ND1 mtDNA, the ratio of ND1 mtDNA and NETs was calculated (ND1 mtDNA/NETs). Differences in mtDNA quantities between the study groups were analyzed by ANOVA, followed by a pairwise *t*-test for the analysis of intergroup differences. The analysis of variations in mtDNA levels across different time points within the same group was performed using the Friedmann test, followed by the pairwise *t*-test for paired groups. For this purpose, septic shock patients were compared with their matched controls. A *p*-value of *p* < 0.05 was considered to be statistically significant. Correlations between mtDNA levels and various parameters were analyzed with Pearson’s correlation coefficient. Experimental data, laboratory routine data, and clinical data were stored in an external database (Microsoft Excel, Redmond, WA, United States). Data were analyzed using R statistical software version 3.6.2 (12 December 2019; www.r-project.org).

## 3. Results

Overall, 80 patients were included (20 patients per study group). Patient characteristics are presented in the primary study, in detail (8). No differences between the four groups were observed for age, sex, or body mass index (BMI) values. The ages of each group, expressed as the median (IQR), were 69 (64–74) for septic shock, 70 (62–79) for CABG, 68 (54–70) for MAS, and 69 (66–74) for CTRL. In the septic shock group, 70% (*n* = 14) were male, while in the CABG group it was 75% (*n* = 15), MAS was 60% (*n* = 12), and CTRL was 70% (*n* = 14). The BMI values were 27.9 (21.7–32.6) for septic shock, 30 (27.6–36.5) for CABG, 24 (22.4–26.9) for MAS, and 27 (23.2–29.2) for CTRL. Regarding the sepsis-related organ failure assessment (SOFA) score, septic shock patients suffered from a severe condition (SOFA onset: 10.5 (10–12.5); 24 h: 11.5 (8–13); 72 h: 9 (5.5–14.5)), leading to an in-hospital mortality of 35% (*n* = 7). Sepsis derived from abdominal origins in 60% (*n* = 12) of cases, whereas a pulmonary or urological source of infection were identified in 15% of cases (*n* = 3), each, and a soft-tissue infection was identified in 10% (*n* = 2) of cases. The MAS group included 40% (*n* = 8) of patients who underwent Whipple’s procedure, open partial colectomy and esophagus resection were each performed in 20% (*n* = 4) of patients, and the remaining patients underwent other types of MAS. The matching of CTRL individuals with septic patients was sufficient to control for pre-existing disease (see details in prior publication [8]).

The anticoagulatory therapy did not differ significantly between the surgical study groups, while septic shock patients received prophylactic heparinization in fewer patients (prophylactic heparinization at onset/24 h/72 h in septic shock, respectively, and preoperative/postoperative/24 h/72 h in surgical patients (% of all patients in each group): septic shock = 50%/60%/55%; CABG = 100%/0%/90%/80%; MAS = 100%/0%/90%/90%; CTRL = 75%). After onset, 24 and 72 h septic shock patients received therapeutic heparinization in 40%, 30%, and 35% of cases, respectively (see details in the primary study [8]).

### 3.1. Quantification of Free-Circulating ND1 mtDNA Plasma Levels

#### 3.1.1. Time Course

**Figure 1:**
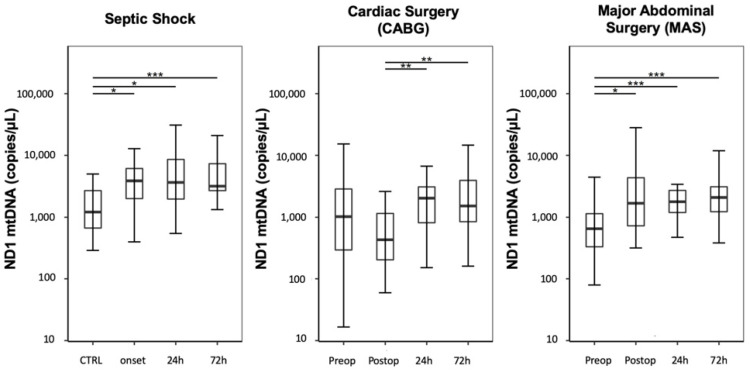
Time course of plasma free-circulating NADH dehydrogenase 1 (ND1) mitochondrial DNA (mtDNA) levels. The results are displayed as boxplot diagrams. Asterisks display the degree of statistical significance: *: *p* < 0.05; **: *p* < 0.01; ***: *p* < 0.001. CTRL: control group.

Compared with the CTRL group, septic shock patients showed a significant increase in ND1 mtDNA plasma levels, expressed as copies/µL, at onset and over three following days (Figure 1; CTRL: 1208 (668–2685); septic shock onset: 3865 (2092–6332); septic shock 24 h: 3650 (1992–9129); septic shock 72 h: 3177 (2680–7318); CTRL vs. septic shock onset: *p* = 0.017; CTRL vs. septic shock 24 h: *p* = 0.013; CTRL vs. septic shock 72 h: *p* < 0.001; all other analyses regarding the analyzed timepoints resulted in *p*-values > 0.05).

Plasma levels of ND1 mtDNA decreased immediately after CABG and increased over the following 72 h (Figure 1; CABG preoperative: 1042 (296–2859); CABG postoperative: 431 (204–1159); CABG 24 h: 2042 (834–3096); CABG 72 h: 1517 (861–3922); CABG postoperative vs. CABG 24 h: *p* = 0.005, CABG postoperative vs. CABG 72 h: *p* = 0.005; all other analyses regarding the analyzed timepoints resulted in *p*-values > 0.05). The preoperative values did not differ from the CTRL group (CABG preoperative vs. CTRL: *p* = 1.0).

In contrast, patients undergoing MAS presented a significant elevation in free-circulating ND1 mtDNA immediately after surgery, followed by a continuous increase over 72 h (Figure 1; MAS preoperative: 650 (333–1139); MAS postoperative: 1716 (727–4346); MAS 24 h: 1780 (1189–2715); MAS 72 h: 2083 (1225–3107); MAS preoperative vs. MAS postoperative *p* = 0.021; MAS preoperative vs. MAS 24 h: *p* < 0.001; MAS preoperative vs. MAS 72 h: *p* < 0.001; all other analyses regarding the timepoints resulted in *p*-values > 0.05). The preoperative values did not differ from the CTRL group (MAS preoperative vs. CTRL: *p* = 1.0).

Compared with post-CABG patients, the free-circulating ND1 mtDNA levels were significantly higher at onset in septic shock patients; however, septic shock levels did not differ significantly compared with those in patients undergoing MAS (septic shock onset vs. CABG postoperative: *p* < 0.001; septic shock onset vs. MAS postoperative: *p* = 1.0; CABG postoperative vs. MAS postoperative: *p* = 0.021).

**Figure 2:**
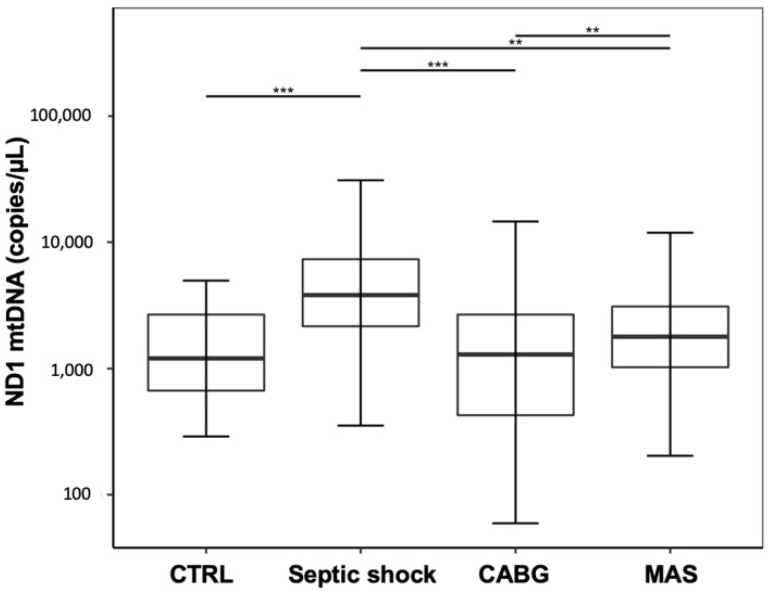
Pooled data analysis for the plasma free-circulating ND1 mtDNA levels for all post-surgical patients, compared with the pooled data for septic patients. Asterisks display the degree of statistical significance: **: *p* < 0.01, ***: *p* < 0.001. Abbreviations: CABG: coronary artery bypass graft; CTRL: control group; MAS: major abdominal surgery.

The pooled data analysis of the combined postsurgical timepoints of each group, respectively of all septic timepoints, revealed significantly higher levels of ND1 mtDNA for septic shock patients compared to CTRL, as well as to both other groups. Nonetheless, the ND1 mtDNA levels in both surgical cohorts were not different from CTRL, but differed significantly from each other, with higher levels in MAS. (Figure 2; CTRL: 1208 (668–2685); septic shock: 3823 (2170–7318); CABG: 1272 (417–2720); MAS: 1356 (694–2845); CTRL vs. septic shock: *p* < 0.001; CTRL vs. CABG: *p* = 0.660; CTRL: vs. MAS: *p* = 0.190; septic shock vs. CABG: *p* < 0.001; septic shock vs. MAS: *p* = 0.006; CABG vs. MAS: *p* = 0.01).

#### 3.1.2. Ratio between Plasma Levels of Free-Circulating ND1 mtDNA and NETs

**Figure 3:**
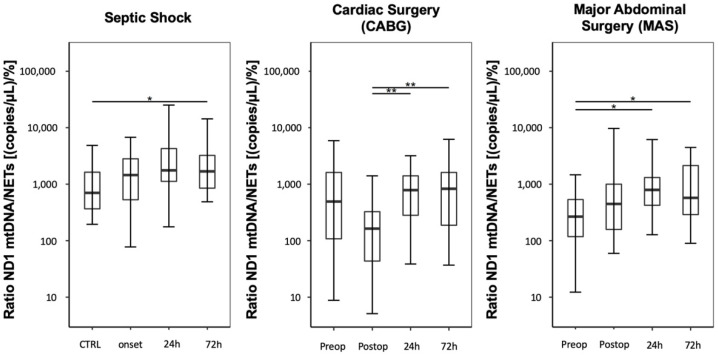
Time course showing the ratio between free-circulating ND1 mtDNA and NETs levels. The results are displayed as boxplot diagrams. Asterisks display the degree of statistical significance: *: *p* < 0.05; **: *p* < 0.01. CTRL: Control group.

Compared with the CTRL group, septic shock patients presented increased ND1 mtDNA/NET ratios, expressed as (copies/µL)/%, starting at onset and lasting over 72 h (Figure 3; CTRL: 708 (368–1684); septic shock onset: 1450 (530–2807); septic shock 24 h: 1762 (1139–4250); septic shock 72 h: 1682 (849–3229)). However, only the measurement after 72 h reached significance, compared with the control group (*p* = 0.021).

Immediately after CABG, patients showed a significant decrease in the mtDNA/NET ratio, followed by increases at 24 and 72 h (Figure 3; CABG preoperative: 494 (109–1669); CABG postoperative: 164 (44–328); CABG, 24 h: 787 (296–1404); CABG, 72 h: 829 (188–1615); CABG postoperative vs. CABG 24 h: *p* = 0.002; CABG postoperative vs. CABG 72 h: *p* = 0.002).

Compared to preoperative values, MAS led to a postsurgical increase in the mtDNA/NET ratio over three days (Figure 3; MAS preoperative: 271 (118–526); MAS postoperative: 452 (160–997); MAS, 24 h: 791 (425–1309); MAS, 72 h: 573 (290–2160); MAS preoperative vs. MAS 24 h: *p* = 0.013; MAS preoperative vs. MAS 72 h: *p* = 0.013).

**Figure 4:**
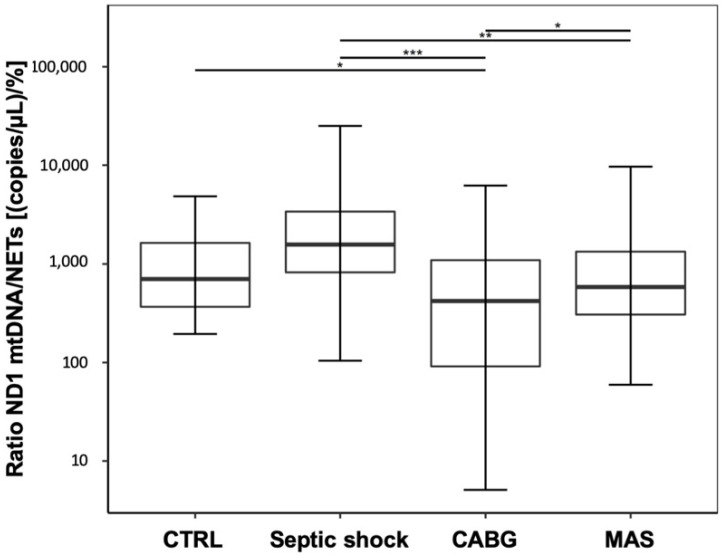
Pooled data analysis for the ratio between free-circulating ND1 mtDNA and NET levels for all post-surgical patients, compared with the pooled data for septic patients. Asterisks display the degree of statistical significance: *: *p* < 0.05, **: *p* < 0.01; ***: *p* < 0.001. Abbreviations: CABG, coronary artery bypass graft; CTRL, control group; MAS, major abdominal surgery.

A pooled data analysis of the postsurgical and septic data regarding the mtDNA/NET ratio revealed a statistically significant decrease in the CABG patients compared to CTRL, and significantly lower values in CABG and MAS patients compared to septic shock. In contrast, the increase in septic shock patients compared to CTRL was not significant. (Figure 4; CTRL: 708 (368–1684); septic shock: 1566 (822–3377); CABG: 487 (94–1180); MAS: 499 (228–1259); CTRL vs. septic shock: *p* = 0.106, CTRL vs. CABG: *p* = 0.042, CTRL vs. MAS: *p* = 0.535, septic shock vs. CABG: *p* < 0.001, septic shock vs. MAS: *p* = 0.003, CABG vs. MAS: *p* = 0.042).

### 3.2. Correlations between Plasma Free-Circulating ND1 mtDNA Levels and Inflammatory and Coagulatory Parameters

The plasma levels of HMGB-1, IL-8, MPO, procalcitonin, and leukocyte counts showed no significant associations with ND1 mtDNA levels within any of the study cohorts (Table 1; details in Supplementary Materials). Only ND1 mtDNA and C-reactive protein (CRP) in patients undergoing CABG displayed a significant correlation (Table 1).

In septic shock patients, the plasma levels of ND1 mtDNA were not consistently associated with any alterations in the coagulatory system, as measured by thromboelastography (Table 2). However, cardiac surgical patients also showed a strong positive correlation between ND1 mtDNA and fibrinogen levels, as with the results of fibrinogen-dependent thromboelastographic assays (INTEM, EXTEM, and FIBTEM MCF; Table 2), and the international normalized ratio (INR) value correlated negatively with the plasma levels of ND1 mtDNA in cardiac surgical patients. Patients undergoing MAS only showed a positive correlation between ND1 mtDNA levels and the INR (Table 2). The platelet aggregometry measurements did not reveal any correlations with the ND1 mtDNA levels in any of the study groups.

## 4. Discussion

The quantification of plasma free-circulating mtDNA levels, particularly ND1 mtDNA, has previously been used to identify sepsis, surgical trauma, and critical illness [10,13,15,27,28,29,30,31,32]. To our knowledge, this is the first study to examine whether they can be used to discriminate sepsis and surgery-induced systemic inflammatory reactions. With this data analysis, subsequent to our previous study on NETs, we were able to show that distinct developments in the detectable levels of ND1 mtDNA occurred that were dependent on the underlying inflammatory trigger. Whereas septic shock patients presented the elevation of ND1 mtDNA levels consistently over 72 h, surgical patients showed different results. The plasma levels of ND1 mtDNA in patients undergoing MAS increased and remained comparable to the elevation observed in septic shock patients, whereas in cardiac surgical patients ND1 mtDNA plasma levels decreased significantly immediately after CABG. The observed drop in ND1 mtDNA plasma levels immediately after cardiopulmonary bypass stands in contrast with the findings of other studies that have investigated the course of free-circulating mtDNA during cardiac surgery. Yet, only the studies by Qin et al. [27,29,30] have examined ND1 mtDNA, whereas human cytochrome B has been targeted more frequently. Because no detailed data regarding the cardiopulmonary bypass technique is available in most studies, the reason for the observed decrease in our study remains unclear, although a dilutional effect might offer an explanation. Moreover, although the absolute ND1 mtDNA level was higher in our study compared with that reported by Qin et al. [29], the increase in the ND1 mtDNA level was not significantly elevated compared with the baseline level. The increase in free-circulating mtDNA is a well-known characteristic of sepsis, and has previously been connected with organ failure and poor prognosis [10,13,15,31,32], but detailed data regarding changes in mtDNA plasma levels are lacking for abdominal surgical patients. Hu et al. [30] revealed an association between the quantities of various mtDNA fragments and the severity of intra-abdominal infections following major trauma. However, these data are not transferable to elective MAS patients, because the studied patients suffered from a trauma-induced severe inflammatory response prior to the surgical procedures. Although mitochondrial haplogroups have been identified as potential biomarkers for the prognosis of sepsis in abdominal surgical patients, the mtDNA level was not quantified in that study [33]. To detect differences between the study groups, we pooled the data from all postsurgical timepoints and compared them with the pooled data from septic patients. This analysis revealed that in comparison with the CTRL group, only septic shock patients showed significantly elevated plasma levels of ND1 mtDNA, whereas the surgical groups did not differ significantly from the CTRL group. Compared to septic shock patients, ND1 mtDNA levels were significantly lower within the surgical patients, most probably caused by the decrease of ND1 mtDNA immediately after cardiopulmonary bypass. This difference was stronger in CABG patients, and was also visible in comparison to the patients who underwent MAS. For this reason, this study supports the hypothesis that the amount of free-circulating plasma ND1 mtDNA levels is potentially helpful for the discrimination of septic shock from surgery-induced systemic responses, and is therefore a promising target for future studies, including larger numbers of patients. Furthermore, ND1 mtDNA could potentially be evaluated in context of a multimarker strategy for identifying postsurgical sepsis. Nonetheless, it has to be considered that the data was scattered with overlapping data ranges, not allowing us to easily define a cut-off value to be used for clinical discrimination between post-surgical inflammation and sepsis. The evaluation of such a threshold might be challenging, and will surely require further studies with larger numbers of patients included.

The primary study upon which the present data analysis was based aimed to quantify NETs using a novel flow cytometry-based method [8]. The interest in examining the additional mtDNA measurements is derived from the hypothesis that free-circulating mtDNA levels might represent a large proportion of NETs. Therefore, we examined the ratio between ND1 mtDNA and NETs, and found comparable time courses between the mtDNA/NET ratio and the ND1 mtDNA plasma levels, indicating that ND1 mtDNA levels likely represent a large proportion of NETs. This finding is supported by the study performed by Yousefi et al. [11], who identified mtDNA release as a pivotal component of vital NETosis. During vital NETosis, neutrophils release mtDNA through the stimulation of TLR-4 and complement factor 5a in a reactive oxygen species-dependent way, resulting in NETs containing solely mtDNA. However, very little in vivo data are available regarding mtDNA-containing NETs, and the available data was primarily derived from trauma patients [3,12,34]. The mtDNA/NET ratio results in our study indicate that mtDNA-containing NETs derived from vital NETosis might play an in vivo role during septic shock and surgery-induced systemic inflammation. However, because mtDNA is also well-known as a damage-associated molecular pattern DAMP that is released during sepsis, the simultaneous release of NETs and mtDNA (independent of vital NETosis) might also explain our findings [3,10,31,32]. Therefore, further research efforts remain necessary to differentiate between mtDNA-containing NETosis and NET-independent mtDNA release.

NETs play a pivotal role in the interaction between the inflammatory and coagulatory system (called immunothrombosis) [16,35]. Especially under septic conditions, NETs can activate platelets and the plasmatic coagulatory system, which is also supported by our previous studies [6,8,16,17,36]. However, except for fibrinogen-dependent parameters in cardiac surgical patients, no consistent associations between the levels of free-circulating ND1 mtDNA and any coagulatory changes were detectable in any of the study groups. On the other hand, this study was not designed for this purpose of investigation. Interestingly, all fibrinogen-dependent thromboelastography assays and the plasma levels of fibrinogen were positively correlated with ND1 mtDNA levels, whereas the INR showed a negative association with ND1 mtDNA levels in cardiac surgical patients, indicating the in vivo pro-coagulatory and fibrinogen-dependent influence of ND1 mtDNA. These results might agree with the findings from Qin et al. Based on the detection of a close relationship between plasma mtDNA levels and activated platelets in cardiac surgical patients, Qin et al. [27] suggested that mtDNA might also be partially derived from activated platelets. Therefore, our findings might describe the in vivo effects of CABG-induced platelet activation, which is strongly associated with fibrinogen-dependent platelet aggregation. On the other hand, it should be mentioned that in patients suffering from coronary artery disease, fibrinogen is also released as an acute phase protein, which might explain the positive correlation of fibrinogen and ND1 mtDNA plasma levels in this particular study group. The same mechanism might also explain the association of CRP and ND1 mtDNA plasma levels in CABG patients. Nonetheless, our findings could support the conclusion by Bhagirath et al. [9], who postulated that free-circulating DNA of any type (including mtDNA) leads to the activation of platelets and thrombin generation. Interestingly, our primary study identified a negative correlation between the detected NET levels and fibrinogen-dependent assays in cardiac surgical patients, indicating that ND1 mtDNA might activate the coagulatory system independent of NETs [8].

Our study features some limitations. First, this secondary analysis represents an observational study, and is not able to draw conclusions regarding the underlying causalities. Second, no sample size calculation was performed regarding either the quantification of ND1 mtDNA or the coagulatory analyses. Since the primary study was designed as a proof-of-concept study aiming to evaluate flow cytometry-based NET quantification, the necessary number of included patients was planned with only 20 patients per group. This might explain our failure to detect further interactions between ND1 mtDNA and inflammatory or coagulatory parameters in the other study groups. In particular, the lack of correlations between inflammatory parameters and ND1 mtDNA levels should be reevaluated using a larger number of patients. However, these findings might have resulted from an increase of the HMGB-1 and MPO levels observed in surgical patients and potentially unidentified influencing factors, such as arteriosclerosis and systemic heparin application, which were highly prevalent in the investigated patients [37,38]. Third, even though all patients received comparable anticoagulatory regimens without intergroup differences (details are presented in the primary study [8]), those regimens’ influence on the performed coagulatory analyses cannot be ruled out. Furthermore, high-dose heparinization throughout cardiopulmonary bypass and its antagonization might have influenced the coagulatory analysis, even though antagonization was effective in terms of activated clotting time. Subsequently, the correlation of fibrinogen and CRP to ND1 mtDNA plasma levels in CABG patients might be triggered by arteriosclerosis, questioning the specificity of ND1 mtDNA plasma levels. Fourth, due to a methodological limitation, PCT values are lacking in the surgical groups. However, among the surgical groups, we did not reveal any postoperative (suspected) infections. That was one explanation for why we did not find PCT values among the post-surgical patients. Lastly, even though pneumonia represents the most common source of sepsis, it was only present in a minor part of the included patients. Due to the high clinical impact of septic shock caused by pneumonia, the role of free-circulating ND1 mtDNA in pneumogenic sepsis should be evaluated in future studies.

## 5. Conclusions

In summary, the herewith presented analysis identifies ND1 mtDNA as a potential biomarker for the discrimination of septic shock and postsurgical systemic inflammation. Plasma levels of free-circulating ND1 mtDNA were significantly higher in septic shock patients compared to all other study groups. Surgical patients showed no increase of ND1 mtDNA plasma levels compared to the CTRL group. These study results indicate that ND1 mtDNA might be useful as a discriminative biomarker for perioperative sepsis. Furthermore, this study shows an association between ND1 mtDNA levels and a fibrinogen-dependent pro-coagulatory shift in cardiac surgical patients.

## Figures and Tables

**Table 1:** Correlations between free-circulating plasma ND1 mtDNA levels and inflammatory parameters. Data were derived from Pearson’s correlation analysis.

Parameters	Septic Shock		Cardiac Surgery (CABG)		Major Abdominal Surgery (MAS)		Control (CTRL)	
	*r*	*p*-Value	*r*	*p*-Value	*r*	*p*-Value	*r*	*p*-Value
MPO (ng/L)	−0.01	0.92	0.09	0.52	0.22	0.09	0.35	0.13
HMGB-1 (pg/L)	−0.14	0.31	−0.05	0.69	0.10	0.45	0.30	0.20
IL-8 (pg/L)	−0.07	0.63	−0.13	0.33	0.22	0.09	−0.02	0.94
NETs (%)	−0.15	0.26	−0.24	0.26	−0.03	0.85	−0.25	0.28
Leucocyte count (L^−1^)	−0.03	0.83	−0.21	0.11	−0.05	0.73	−0.30	0.20
CRP (mg/L)	0.17	0.22	0.44	*<0.001*	−0.01	0.92	−0.05	0.84
PCT (µg/L)	0.03	0.82	N.A.	N.A.	0.23	0.43	N.A.	N.A.

Significant *p*-values are highlighted in italics. Abbreviations: CABG: coronary artery bypass graft; CRP: C-reactive protein; CTRL: control group; HMGB1: high mobility group protein B1; IL-8: interleukin 8; MAS: major abdominal surgery; MPO: Myeloperoxidase; NETs: neutrophil extracellular traps; N.A.: not available; PCT: procalcitonin.

**Table 2:** Correlation of free-circulating plasma levels of ND1 mtDNA to coagulatory parameters. Data were derived from Pearson’s correlation analysis.

Parameters	Septic Shock		Cardiac Surgery (CABG)		Major Abdominal Surgery (MAS)		Control (CTRL)	
	*r*	*p*-Value	*r*	*p*-Value	*r*	*p*-Value	*r*	*p*-Value
Thromboelastography								
EXTEM CT (s)	0.01	0.96	−0.19	0.15	−0.33	*0.01*	0.15	0.54
INTEM CT (s)	0.10	0.48	−0.16	0.23	−0.10	0.47	−0.16	0.51
FIBTEM CT (s)	0.15	0.27	−0.05	0.71	−0.39	*<0.001*	0.16	0.50
NATEM CT (s)	0.20	0.14	−0.01	0.91	−0.13	0.33	0.01	0.96
EXTEM CFT (s)	−0.11	0.41	−0.18	0.19	0.29	*0.03*	0.52	*0.02*
INTEM CFT (s)	−0.09	0.53	−0.18	0.19	0.03	0.84	0.32	0.16
FIBTEM CFT (s)	−0.11	0.42	−0.08	0.63	−0.10	0.51	0.29	0.36
NATEM CFT (s)	0.17	0.23	0.23	0.08	−0.12	0.39	−0.18	0.44
EXTEM MCF (mm)	0.07	0.60	0.35	*0.01*	−0.20	0.14	−0.32	0.18
INTEM MCF (mm)	0.08	0.55	0.31	*0.02*	−0.17	0.21	−0.26	0.26
FIBTEM MCF (mm)	0.05	0.74	0.46	*<0.001*	−0.13	0.35	−0.12	0.62
NATEM MCF (mm)	−0.42	*<0.001*	0.24	0.08	0.03	0.82	−0.09	0.72
EXTEM LI60 (%)	0.08	0.55	*0.01*	0.93	0.28	*0.04*	0.07	0.76
INTEM LI60 (%)	0.06	0.64	−0.03	0.80	0.31	*0.02*	0.06	0.79
FIBTEM LI60 (%)	0.08	0.55	0.10	0.45	0.11	0.42	−0.12	0.63
NATEM LI60 (%)	0.25	0.09	0.07	0.61	0.14	0.31	0.06	0.81
Impedance Aggregometry								
ASPI (Units)	0.00	0.97	−0.06	0.64	−0.21	0.12	0.29	0.21
ADP (Units)	0.06	0.66	0.01	0.92	−0.23	0.09	0.11	0.65
TRAP (Units)	−0.01	0.93	−0.03	0.84	−0.19	0.16	0.29	0.22
Global coagulatory Parameters								
Platelet count (L^−1^)	0.01	0.96	−0.18	0.18	−0.11	0.42	−0.28	0.23
PTT (s)	0.09	0.53	−0.20	0.13	0.23	0.11	0.08	0.75
INR	0.07	0.63	−0.37	*<0.001*	0.30	*0.03*	−0.17	0.49
Fibrinogen (g/L)	0.12	0.54	0.57	*<0.001*	0.03	0.89	N.A.	N.A.

Significant *p*-values are highlighted in italics. Abbreviations: CABG: coronary artery bypass graft; CFT: clot firmness time; CT: clotting time; CTRL: control group; INR: international normalized ratio; LI60: lysis index after 60 min; MAS: major abdominal surgery; MCF: mean clot firmness; N.A.: not available; PTT: partial thromboplastin time.

## Supplementary Materials

The following can be found online at https://www.mdpi.com/2077-0383/9/7/2056/s1, Table S1: The results of the inflammatory parameters as presented in [8]. Data are shown as medians (IQR). Abbreviations: CRP: C-Reactive Protein; DNA: Deoxynucleic Acid; HMGB1: high mobility group protein B1; MPO: myeloperoxidase; NETs: neutrophil extracellular traps; PCT: procalcitonin.
