# Supplementary material for: Blood Levels of Free-Circulating Mitochondrial DNA in Septic Shock and Postsurgical Systemic Inflammation and Its Influence on Coagulation: A Secondary Analysis of a Prospective Observational Study
Source: J Clin Med. 2020 Jun 30;9(7):2056. doi: 10.3390/jcm9072056 (PMC7408641; doi:10.3390/jcm9072056)
Supplement: Supplementary file 1 [file jcm-09-02056-s001.pdf]

**Supplementary Table 1:** The results of the inflammatory parameters as presented in [8].

| | Septic Shock [*n* = 20] | | Cardiac Surgery [CABG, *n* = 20] | | Major Abdominal Surgery [MAS, *n* = 20] | | Control Patients [CTRL, *n* = 20] | |
|---|---|---|---|---|---|---|---|---|
| **Leucocytes ($L^{-1}$)** | onset | 11.9 [7.1–19.7] | Preop | 8.1 [6.6–9.4] | Preop | 7.6 [6–9] | Ctrl | 5.9 [5.3–7.9] |
| | 24 h | 13.5 [9.3–20.9] | Postop | 11 [7.9–15] | Postop | 10.3 [9.4–12.5] | | |
| | 72 h | 14.2 [10.7–17.3] | 24 h | 10.7 [8.2–12.2] | 24 h | 11.5 [9.3–12.9] | | |
| | | | 72– h | 10.6 [8.2–11.8] | 72 h | 7.4 [6.5–11.6] | | |
| **CRP (mg x $L^{-1}$)** | onset | 229.5 [117.2–277.3] | Preop | 3.8 [1.9–10.6] | Preop | 5.1 [1.7–10.3] | Ctrl | 1.1 [0–6.4] |
| | 24 h | 244.6 [166.5–287.7] | Postop | 4.3 [2.6–9.2] | Postop | 6.5 [2.5–11.4] | | |
| | 72 h | 236.5 [139.5–268.8] | 24 h | 75.1 [67.2–109.8] | 24 h | 68 [46.6–88.5] | | |
| | | | 72 h | 202.4 [156.3–241.2] | 72 h | 149 [115.7–200] | | |
| **PCT (µg x $L^{-1}$)** | onset | 9.2 [5.2–38.1] | Preop | 0.2 [0.1–0.2] | Preop | N.A. | Ctrl | N.A. |
| | 24 h | 10.4 [4.9–29.2] | Postop | N.A. | Postop | 0.6 [0.4–0.7] | | |
| | 72 h | 7 [2.2–25.6] | 24 h | N.A | 24 h | 0.7 [0.3–0.9] | | |
| | | | 72 h | 1.6 [1.6] | 72 h | 0.8 [0.4–0.9] | | |
| **NETs (%)** | onset | 3.2 [2.3–4.2] | Preop | 2 [1.7–2.6] | Preop | 2.6 [1.7–3.3] | Ctrl | 1.6 [1–2] |
| | 24 h | 2.5 [1.8–3.7] | Postop | 3.5 [2.7–4.6] | Postop | 2.9 [2.3–5.2] | | |
| | 72 h | 2.3 [1–3.8] | 24 h | 2.7 [2.1–3.5] | 24 h | 2.6 [2–3.8] | | |
| | | | 72 h | 2.8 [2.1 –3.8] | 72 h | 2.7 [2.3–3.9] | | |
| **HMGB1 (pg x $mL^{-1}$)** | onset | 40,332.1 [25,079.6–51,674.9] | Preop | 25,241.3 [20,953.1–46,031.4] | Preop | 31,126.8 [20,032.8–38,097.8] | Ctrl | 26,297.5 [22,149.3–34,710.9] |
| | 24 h | 32,692.3 [21,563.6–50,421.8] | Postop | 23,982.5 [17,353.2–49,133.1] | Postop | 25,343.5 [21,913.1–41,784.2] | | |
| | 72 h | 25,496.2 [23,125.4–33,421.3] | 24 h | 30,440.2 [22,238.5–41,098.5] | 24 h | 28,800.1 [21,687.7–39,665.6] | | |
| | | | 72 h | 26,584.3 [20,870.2–38,988.1] | 72 h | 21,780.6 [16,867–34,755.6] | | |
| **MPO (ng x $mL^{-1}$)** | onset | 700,905.7 [285,135.5–886,644] | Preop | 392,102.8 [199,581–571,528,04] | Preop | 367,381.5 [187,582–499,310.8] | Ctrl | 214,472.6 [136,124.2–296,626.7] |
| | 24 h | 542,611.2 [303,891–832,728.9] | Postop | 438,502.8 [341,657.5–638,995.4] | Postop | 480,111 [344,182.5–885,513.8] | | |
| | 72 h | 498,553 [381,058.9–610,573.3] | 24 h | 595,820.4 [275,593.4–892,010.7] | 24 h | 713,023.1 [433,356.9–913,219.4] | | |
| | | | 72 h | 529,317.3 [306,869.6–885,046] | 72 h | 351,888,.5 [235,179.9–711,455.7] | | |
| **Interleukin 8 (pg x $mL^{-1}$)** | onset | 470.4 [105.9–1462,30] | Preop | 39.2 [26.1–49] | Preop | 35 [20.4–49.8] | Ctrl | 35.8 [25–40.5] |
| | 24 h | 206.6 [100.1–489.9] | Postop | 85.3 [57.7–127.9 | Postop | 71.1 [58.2–129.1] | | |
| | 72 h | 165.1 [90.2–195.5] | 24 h | 67.1 [40.7–99] | 24 h | 60.9 [41.2–110.4] | | |
| | | | 72 h | 55.2 [42.9–72.2] | 72 h | 41.9 [27.1–63.6] | | |

Data are shown as medians (IQR). CRP: C–Reactive Protein; DNA: Deoxynucleic Acid; HMGB1: high mobility group protein B1; MPO: myeloperoxidase; NETs: neutrophil extracellular traps; PCT: procalcitonin.
